# Online Mental Health Assessments of COVID-19 Patients in South Korea

**DOI:** 10.3389/fpsyt.2021.685445

**Published:** 2021-07-06

**Authors:** Jung Hyun Lee, Dayoung Lee, Soyoen Hyun, Ji Sun Hong, Chang-Hoon Kim, Woojin Kim, Minyoung Sim

**Affiliations:** Division of Disaster Mental Health Services, National Center for Mental Health, Seoul, South Korea

**Keywords:** COVID-19, post-traumatic stress disorder, depression, anxiety, psychological trauma

## Abstract

Experiences of infectious diseases cause stressful and traumatic life events, hence, coronavirus disease 2019 (COVID-19) patients could suffer from various mental health problems requiring psychological support services. This study investigates the severity of mental health problems among confirmed COVID-19 patients. From March to November 2020, we collected the data from 118 COVID-19 patients who voluntarily participated in the National Center for Disaster Trauma's online mental health assessment consisting of self-report scales like Primary Care of Posttraumatic Stress Disorder screen (PC-PTSD), Generalized Anxiety Disorder-7 (GAD-7), Patient Health Questionnaire-9 (PHQ-9), Patient Health Questionnaire-15 (PHQ-15), and P4 Suicidality Screener. For control, 116 other disaster-experienced and 386 non-COVID-19-experienced participants were recruited. The COVID-19 patients showed more severe symptoms including post-traumatic symptoms, depression, anxiety, and somatic symptoms than control groups across all four screening scales (*p* < 0.001). Regarding high-risk, COVID-19 patients had an increased association with high-risk compared to the comparison groups (PC-PTSD: OR = 24.16, 95% CI = 13.52–43.16 *p* < 0.001; PHQ-9: OR = 14.45, 95% CI = 8.29–25.19, *p* < 0.001; GAD-7: OR=20.71, 95% CI = 10.74–39.96, *p* < 0.001; PHQ-15: OR = 5.65, 95% CI = 3.44–9.25, *p* < 0.001; P4: OR = 14.67, 95% CI = 8.95–25.07, *p* < 0.001). This study's results imply that there is a high-risk of overall mental health problems, especially stronger associations of post-traumatic stress symptoms, in COVID-19 patients. These findings help inform practitioners about the psychological responses to COVID-19 experiences and to prepare appropriate interventions and services for the incremental number of confirmed cases.

## Introduction

The coronavirus disease 2019 (COVID-19) outbreak is one of the largest pandemic disasters of this century. This disaster started in December 2019 and the World Health Organization (WHO) declared it a pandemic on 11^th^ March 2020 (1). An outbreak of COVID-19 has continued to worsen in Korea since the first confirmed patient was reported on January 2020. The need for mental health services during pandemics and other disasters was emphasized during the Middle East respiratory syndrome coronavirus (MERS-nCOV) outbreak in 2015, Therefore, multidisciplinary psychosocial support has been provided since the early stages of the outbreak in South Korea (2). The manifestation of COVID-19 varies from no symptoms to severe acute respiratory distress and high fatality (3). It is suggested that patients with COVID-19 may struggle with both, life-threatening fear of the infection and quarantine-related stressors that require psychological support during this pandemic (4, 5). However, there is little empirical evidence regarding COVID-19 survivors' mental health.

The outbreak of severe acute respiratory syndrome (SARS) gives us the most recent data on mental health problems related to infectious disease. Among SARS survivors who were discharged from the hospital for 4 weeks, about 32 and 27% of participants were classified as having over “moderate” ranges of anxiety and depressive symptoms, respectively, which were higher than community samples (6). Regarding the long-term psychiatric morbidities among SARS survivors, 25.6% of the patients had post-traumatic stress disorder (PTSD) and 15.6% had depressive disorders 30 months after the SARS outbreak (7). A systematic review of coronavirus, including SARS, MERS-nCOV, and COVID-19, reported reduced health-related quality of life (HRQol) in survivors (8). Additionally, 38.8% of them experienced post-traumatic stress disorder (PTSD), 33.2%, depression, and 30.0%, anxiety after 6 months (8). Previous studies suggested that a substantial portion of survivors suffered from psychiatric symptoms in either early or late aftermath of SARS. Besides physical symptoms, patients with infectious diseases experienced various stressors such as isolation, fear transmitting the virus to others, and social stigma which may lead them to experience psychological distress, loneliness, anxiety, depression, and post-traumatic stress symptoms (PTSS) (5, 9).

Most of the research related to COVID-19 and mental health has focused on the mental health of quarantined individuals and the general population. However, currently, there are only a few studies that have examined COVID-19 patients' mental health (10, 11). A study reported a high prevalence of PTSD and related risk factors in patients after severe COVID-19 (11). Furthermore, a recent retrospective cohort study reported increased risks of psychiatric and neurological morbidity in patients, six months after COVID-19 infection (12). Thus, an updated study on in COVID-19 patients' mental health problems is required to provide timely therapeutic approaches and mental health services. The National Center for Disaster Trauma (NCT) in South Korea was put in charge of psychological support services for COVID-19 patients. The NCT provides self-rated online assessments to encourage COVID-19 patients to use mental health services if needed. Self-rated assessments can provide valuable information to understand confirmed patients' psychological responses.

This study investigates confirmed COVID-19 patients' mental health, especially those who voluntarily seek mental health support. We hypothesized that COVID-19 patients would suffer from more severe symptoms including PTSS as well as depression, anxiety, somatic symptoms, and suicidality compared to those who did not have COVID-19.

## Methods

### Data Collection

In Korea, the Integrated Psychological Support Group for COVID-19 under the Ministry of Health and Welfare has been set up to take charge of mental health services amid the COVID-19 outbreak from 29^th^ January 2020 till date (2). National Center for Disaster Trauma (NCT) has a main role in managing this governmental organization and has provided psychological support service via a 24-h hotline for COVID-19 patients, quarantined individuals, and their families. To provide information, including stress management, available mental health services, and utility and online screening tool for mental health, we sent the text-messages to the list of confirmed patients given by the government. Additionally, individuals who visited either the official NCT website or social network services could also participate in the online mental health screening. When patients accessed the website, they were informed, “It is normal to feel any distress during or after COVID-19 treatment and quarantine, and it would be helpful to check one's psychological distress with the following online mental health assessment. Based on the result of the assessment, we will contact to you and provide psychological support services.” Those who agreed and provided informed consent could start answering the questionnaires: demographic information (age and sex) and five self-reported scales. After completing the questionnaires they could find the total score for each test. From March to November 2020, 118 confirmed patients responded to the online mental health assessment, in this study.

As a comparison group, individuals who had not experienced COVID-19 were recruited and data from participants who participated in disaster mental health programs by the NCT, which provided regular education such as Psychological First Aid to the general public and mental health-related workers from 2018 to 2019, were used. Prior to the education, participants were asked to complete surveys, including the life-time experiences of disaster and mental health assessments, comprising the same scales as COVID-19 patients. A total of 492 participants voluntarily responded to the survey and were classified in disaster-experienced and -inexperienced groups in this study.

The present study was conducted as a part of the NCT research project, which was reviewed and approved by the Institutional Review Board of NCMH (approval No.116271-2020-29).

### Mental Health Assessment Tools

The online mental health assessment comprised five screening scales including Primary Care of Posttraumatic Stress Disorder screen for DSM-5 (PC-PTSD), Generalized Anxiety Disorder-7 (GAD-7), Patient Health Questionnaire-9 (PHQ-9), Patient Health Questionnaire-15 (PHQ-15), and P4 Suicidality Screener.

The PC-PTSD was used to assess the PTSS that were experienced during the last month (13, 14). Some of those in disaster-experienced and disaster-inexperienced group, who participated from 2018 to March 2019, completed the PC-PTSD-4, meanwhile confirmed patients and those in the disaster-experienced group, who participated after March 2019, completed the PC-PTSD-5 (15). Participants answered “1: yes” or “0: no” for each item, and the severity of PTSS was classified based on the total score of each item such as 0–1: normal, 2: mild-severe and higher than 3: severe. Preliminary results from validation studies suggest a cut-point of 3 (13). PC-PTSD score was aggregated from 4 items which were common in PC-PTSD-4 and PC-PTSD-5. Based on the cut-off point of 3, we classified participants into “Above cut-off” and “Below cut-off” groups. Cronbach's alpha for 4 items used to aggregate PC-PTSD score was 0.76 in this study.

Patient Health Questionnaire (PHQ-9) was used to assess depression in the last 2 weeks (16). The PHQ-9 comprises nine items rated on 4-point Likert scale (0: *Not at all*−3: *Nearly every day*). The severity of depressive symptoms is classified into the following five groups: 0–4 (None), 5–9 (Mild), 10–14 (Moderate), 15–19 (Moderately severe), and over 20 (Severe); the suggested cutoff point is 10. Participants with a total score over 10 were classified as the high-risk group and those with scores under 10, as the low-risk group. Cronbach's alpha for the PHQ-9 was 0.88 in this study.

Generalized Anxiety Disorder-7 (GAD-7) was used to assess anxiety symptoms in the previous 2 weeks (17). The GAD-7 comprises seven items rated on a 4-point Likert scale (0: *Not at all*−3: *Nearly every day*). The severity of the anxiety symptoms is classified into the four following groups: 0–4 (Normal), 5–9 (Mild), 10–14 (Moderate), and over 15 (Severe). A high-risk group comprised of those who received over 10 points (17). Cronbach's alpha for the GAD-7 was 0.89 in this study.

Patient Health Questionnaire-15 (PHQ-15) was used to assess somatic symptoms in the last month (18). The PHQ-15 consists of 15 items rated on 3-point Likert scale (0: *Not bothered at all*−2: *Bothered a lot*). The severity of somatic symptoms is classified into the following four groups: 0–4 (Normal), 5–9 (Mild), 10–14 (Moderate), and over 15 (Severe); the suggested cutoff point is 10. Participants with a total score over 15 were classified as the high-risk group. Cronbach's alpha for the PHQ-15 was 0.86 in this study.

The P4 Screener was used to assess potential suicide risk (19). It consists of four items: “(1) Have you ever attempted to harm yourself in the past?,” “(2) Have you thought about how you might actually hurt yourself?,” “(3) How likely do you think it is that you will act on these thoughts about hurting yourself or ending your life some time over the next month?,” and “(4) Is there anything that would prevent or keep you from harming yourself?” If the answer is not “Yes” for the items (1) and (2), the participant is classified as at “Minimal” risk. If the participants chose “Somewhat likely” or “Very likely” for item (3), or “No” for item (4), they were classified as “Higher (high-risk group),” while the others were classified as “Lower (low-risk group).”

### Statistical Analysis

To examine group differences among COVID-19 patients and disaster-experienced and -inexperienced individuals, demographic and clinical characteristics were analyzed using χ^2^ test for categorical variables and analysis of variance (ANOVA) for continuous variables. Comparison of each mental health assessment was performed using ANOVA with Tukey's HSD *post-hoc* test. We perform a Pearson correlation analysis to determine the statistic relationship between each of the mental-health-related variables. Additionally, multivariate logistic regression was used to explore the association among COVID-19 patients' moderate to severe symptoms and the comparison groups. The “Above cut-off” group of each scale was used as the outcome variable. Then, the odds ratio for the “Above cut-off” group among COVID-19 patients was calculated using covariates, including age and sex. The significance level was set at *p* < 0.05. Statistical analyses were conducted using the statistical package R version 4.0.2 for Windows.

## Results

A total of 118 COVID-19 patients participated in the online mental health assessment. Of which, 34 (28.8%) were male and 84 (71.4%) were female. The mean age of COVID-19 patients in the online mental health assessment was 32.69 (SD = 13.59), which was lower than that of the disaster-experienced and -inexperienced groups {*F*_(2,605)_ = 36.08, *p* < 0.001}. The result of ANOVA indicated that there were statistically significant differences in all mental health assessment scores {(PC-PTSD, *F*_(2,604)_ = 102.4, *p* < 0.001; PHQ-9: *F*_(2,604)_ = 99.36, *p* < 0.001; GAD-7, *F*_(2,605)_ = 147.9, *p* < 0.001; PHQ-15: *F*_(2,53.98)_ = 59.98, *p* < 0.001) In the *post-hoc* analysis, COVID-19 patients showed higher total score in all measurements compared to the other groups (Table 1). Among the COVID-19 patients, the mental health assessments were significantly correlated with each other (*p* < 0.001), with correlation coefficients ranging from 0.44 to 0.86 (Table 2). The PHQ-9 was the most strongly correlated with the GAD-7 (*r* = 0.86); it was also strongly correlated with the PC-PTSD and the PHQ-9 (*r* = 0.67 and *r* = 0.68, respectively). There were moderate correlations between the PC-PTSD and the GAD-7 and the PC-PTSD and the PHQ-15 (*r* = 0.59 and *r* = 0.44, respectively).

**Table 1:** Demographic and clinical characteristics of the participants.

	**COVID-19 Patients (A)**	**Disaster-experienced (B)**	**Disaster-inexperienced (C)**	***X*^**2**^/F**	***Post-hoc* Tukey HSD**	***p***
	**(*N* = 118)**	**(*N* = 116)**	**(*N* = 386)**			
Age	32.69 ± 13.59	41.04 ± 16.15	43.88 ± 10.67	*F*_(2,605)_ = 36.08	A < B, A < C	<0.001
Sex				$X_{\left(2\right)}^{2}$ = 15.04		
Males	34 (28.8%)	28 (24.3%)	159 (41.8%)			<0.001
Females	84 (71.2%)	87 (75.7%)	221 (58.2%)			
PC-PTSD	2.5 ± 1.4	1.0 ± 1.3	0.7 ± 1.1	*F*_(2,604)_ = 102.40	A>B>C	<0.001
PHQ9	11.21 ± 6.68	4.50 ± 5.32	4.03 ± 4.04	*F*_(2,604)_ = 99.36	A>B, A>C	<0.001
GAD	9.24 ± 5.60	2.85 ± 3.86	2.40 ± 3.06	*F*_(2,583)_ = 147.90	A>B, A>C	<0.001
PHQ15	10.53 ± 5.57	6.59 ± 5.40	5.16 ± 4.47	*F*_(2,605)_ = 53.98	A>B>C	<0.001
P4						
Low risk	106 (89.8%)	110 (98.2%)	376 (99.5%)	$X_{\left(2\right)}^{2}$ = 33.00	A>B>C	<0.001
High risk	12 (10.2%)	2 (1.8%)	2 (0.5%)			

*PC-PCSD, Primary Care PTSD Screen; PHQ-9, Patient Health Questionnaire-9; GAD-7, Generalized Anxiety Disorder; Screener P4, P4 suicidality Screener. PC-PTSD score was calculated by summing the 4 items that were common in PC-PTSD-4 and PC-PTSD-5*.

**Table 2:** Pearson correlation matrix for mental-health-related variables in COVID-19 patients (*n* = 118).

**Variables**	**1**	**2**	**3**	**4**
1. PC-PTSD	1	-	-	-
2. PHQ-9	0.67^*^	1	-	-
3. GAD-7	0.59^*^	0.86^*^	1	-
4. PHQ-15	0.44^*^	0.68^*^	0.65^*^	1

*PC-PCSD, Primary Care PTSD Screen; PHQ-9, Patient Health Questionnaire-9; GAD-7, Generalized Anxiety Disorder; Screener P4, P4 suicidality Screener.*

* *p < 0.01*.

**Figure 1:**
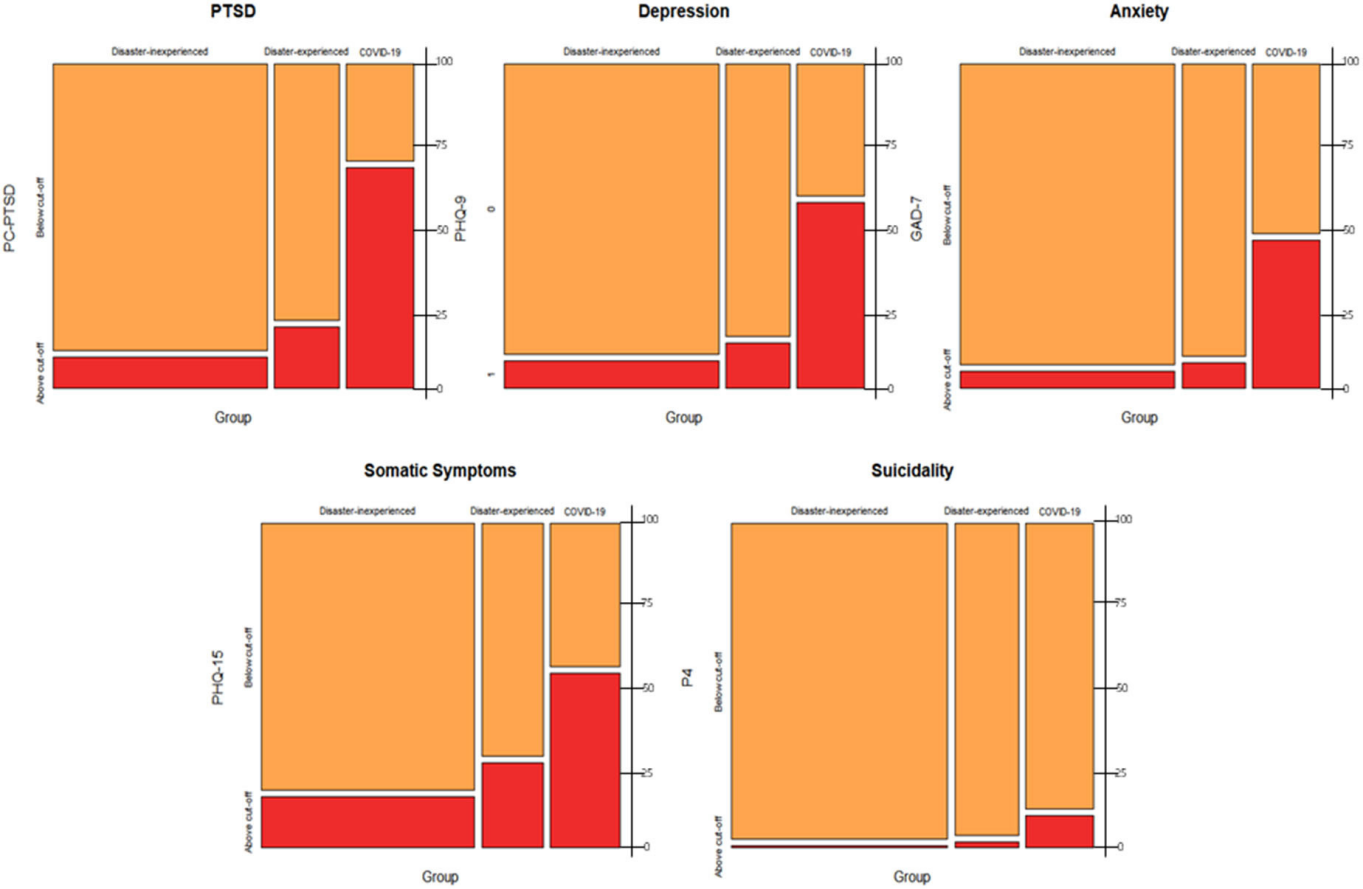
Mosaic plots of severe mental health problems among COVID-19 patients and comparison groups. Mosaic plot depicting that the proportion of “above cut-off” group (red) for each of the mental-health-related variables in COVID-19 patients is significantly greater than that in the control groups. PC-PCSD, Primary Care PTSD Screen; PHQ-9, Patient Health Questionnaire-9; GAD-7, Generalized Anxiety Disorder; Screener P4, P4 suicidality Screener.

The proportions of “Above cut-off” for each assessment were significantly different in COVID-19 patients and disaster-experienced and -inexperienced groups (Figure 1, PC-PTSD, $\chi_{\left(2\right)}^{2}$ =123.75, *p* < 0.001; PHQ-9, $\chi_{\left(2\right)}^{2}$ = 88.78, *p* < 0.001; GAD-7, $\chi_{\left(2\right)}^{2}$ = 160.42, *p* < 0.001; PHQ-15, $\chi_{\left(2\right)}^{2}$ = 53.96, *p* < 0.001).

The result of logistic regression suggested that, COVID-19 patients had higher adjusted odds ratios (AOR) for “Above cut-off” across all measurements compared to disaster-inexperienced group (PC-PTSD, AOR = 24.14, 95% CI = 13.52–43.16; PHQ-9, AOR = 14.45, 95% CI = 8.29–25.19; GAD-7, AOR = 20.71, 95% CI = 10.74–39.96; PHQ-15, AOR = 5.65, 95%CI = 3.44–9.25; P4, AOR = 14.67, 95% CI = 8.95–25.07). The COVID-19 patients had stronger associations with significant AORs for PC-PTSD and PHQ-15 compared to disaster-experience group (Table 3).

**Table 3:** Adjusted odds ratio of severe mental health problems among COVID-19 patients and comparison groups.

	**Disaster-inexperienced Group (*****n*** **=** **386)**		**Disaster-experienced Group (*****n*** **=** **116)**		**COVID-19 patients** **(*****n*** **=** **118)**	
	***n* (%)**	**OR** **(95% CI)**	***n* (%)**	**OR** **(95% CI)**	***n* (%)**	**OR** **(95% CI)**
PC-PTSD						
Below cut-off	339 (90.2%)		92 (80.7%)		36 (30.5%)	
Above cut-off	37 (9.8%)	1.0	22 (19.3%)	2.02 (1.11–3.67)^*^	82 (69.5%)	24.16 (13.52–43.16)^**^
PHQ-9						
Below cut-off	344 (91.2%)		96 (85.7%)		49 (41.5%)	
Above cut-off	33 (8.8%)	1.0	16 (14.3%)	1.71 (0.89–3.27)	69 (58.5%)	14.45 (8.29–25.19)^**^
GAD-7						
Below cut-off	358 (94.7%)		103 (92.0%)		63 (53.4%)	
Above cut-off	20 (5.3%)	1.0	9 (8.0%)	1.65 (0.72–3.80)	55 (46.6%)	20.71 (10.74–39.96)^**^
PHQ-15						
Below cut-off	305 (84.0%)		77 (73.3%)		53 (44.9%)	
Above cut-off	58 (16.0%)	1.0	28 (26.7%)	1.73 (1.02–2.94)^*^	65 (55.1%)	5.65 (3.44–9.25)^**^
P4						
Below cut-off	376 (99.5%)		110 (98.2%)		106 (89.8%)	
Above cut-off	2 (0.5%)	1.0	2 (1.8%)	1.11 (0.64–1.93)	12 (10.2%)	14.67 (8.95–25.07)^**^

*PC-PCSD, Primary Care PTSD Screen; PHQ-9, Patient Health Questionnaire-9; GAD-7, Generalized Anxiety Disorder; Screener P4, P4 suicidality Screener.*

*Odds ratios were adjusted for covariates including age and sex.*

* *p < 0.05,*

** *p < 0.001*.

## Discussion

This study provides empirical data on mental health problems related to COVID-19. We found that more than half the COVID-19 patients who participated in the online mental health assessment experienced post-traumatic stress, depression, anxiety, and somatic symptoms. Additionally, one in ten was in the high-risk group for suicidality. There is a paucity of knowledge regarding COVID-19's impact on mental health. Until now, despite the increased concerns about COVID-19 infection as a stressful event, research that evaluates the mental health problems among COVID-19 patients is scarce. In our study, COVID-19 patients reported more severe symptoms including PTSS, depression, anxiety, somatic symptoms, and suicidality compared to the comparison groups who never experienced COVID-19. Additionally, compared to other disaster-experienced individuals from the comparison group, COVID-19 patients received higher scores on all mental health assessments. The “Above cut-off” group, showed greater odds ratios PTSS, depression, somatic symptoms, and suicidality in COVID-19 patients compared to the disaster-inexperienced group. Remarkably, these associations were stronger than those in the disaster-experienced group.

Consistent with the results of previous large-scale pandemic studies, we found that COVID-19 patients have significantly higher rates of mental health problems than the control groups. Existing studies report an alarming proportion (~40%) of SARS or MERS survivors having experienced psychiatric illnesses (14, 20). A long-term follow-up study reported that 45% of participants experienced at least one more psychiatric disorder event after discharge and 58.9% cumulative incidence of psychiatric disorder in the post-SARS era (7). The evidence found in this study points to a strong association between COVID-19 and adverse mental health issues, compared with the control groups. This is in agreement with a previous finding of increased hazard ratios of anxiety and mood disorders in COVID-19 survivors (12, 21). PTSD is commonly seen in those who have survived disasters (22, 23). In our sample, PTSS were most common, with more than two thirds of the COVID-19 patients reporting an “above cut-off” score on the PC-PTSD scale. Additionally, the odds ratio of PTSS was greater than that of other mental health problems in the COVID-19 patients. The likelihood of severe PTSS was 24 times higher in the COVID-19 patients group compared to the control group, which is two-fold higher than the odds ratio of other disaster-experienced groups. Consistent with the definition of traumatic events as exposure to death, threatened death, actual or threatened serious injury, the experience of COVID-19 infection may be sufficient to cause life-threatening fear and helplessness (24). Patients with infectious diseases are likely to experience post-traumatic stress responses due to the traumatic course of infection, including treatment, quarantine, and social stigma (25). Former reports with a high prevalence of PTSD among SARS survivors have been supporting our results. Mak et al. (7) reported that about half of the patients experienced PTSD after the SARS outbreak, and 25% of the patients had been diagnosed with PTSD through clinical interviews even 30-months post-SARS; half of them had a PTSD diagnosis after the SARS outbreak. A study, conducted in China, reported that over 95% of COVID-19 patients who completed an online assessment were found to have PTSS (10). These rates are notably higher than those of SARS survivors. Through a meta-analysis study it was found that the prevalence of PTSD was 32.2%, which was two times higher than depression and anxiety disorder, in the post-illness stage among COVID-19 patients (23). The most recent study, which used the Clinically-Administered PTSD Scale for COVID-19 patients, found that that the prevalence of PTSD was 30.2%, which is greater than previous reports of other types of disasters, such as earthquakes or the World Trade Center disaster (11). Regarding trauma-related symptoms, 75% of Ebola survivors reported re-experience or arousal (26). Since the PC-PTSD is a screening tool for PTSD, clinical diagnosis cannot be awarded for scores above the cut-off; the figures in our sample may have been exaggerated compared to those in previous studies. However, our findings imply that a more individuals may have suffered from PTSS after COVID-19 infection compared to other disasters, which is consistent with a previous study (11). It is therefore essential to provide timely management to alleviate PTSS for COVID-19 patients.

Regarding depression and anxiety, approximately half our participants were classified into higher cut-off groups and had a greater likelihood of mental health issues, compared to the control groups. Previous reports of SARS showed that 10–40% of patients reported over moderate levels of anxiety and depression (7), in the immediate aftermath of SARS. A recent survey on the general population's mental health after the COVID-19 pandemic in South Korea showed that 20.0 and 16.3% of participants scored above the cut-off scores on PHQ-9 and GAD-7, respectively (27). Although direct comparison between our study's findings and with these data is limited, the proportions of confirmed patients in the “Above cut-off” groups, in our study, was greater, with 58.5% for PHQ-9 and 46.6% for GAD-7.

Interestingly, somatic symptoms were also more frequent in COVID-19 patients than in the control group. Somatic symptoms might be directly related to COVID-19 infection or occur because of psychological distress. Trauma or stressors can impair the autonomic nervous system or the stress response system (28), causing somatization or non-specific physical symptoms. Physical symptoms, such as pain, fatigue, and general weakness, make it harder for respiratory infection survivors to return to their work and normal life (29–31). Post-SARS patients complained of fatigue, myalgia, and weakness accompanied by depression and sleep disturbances (30). Vittori et al. (32) suggest a multidisciplinary approach to identify physical and psychological disabilities and long-term effects in survivors of COVD-19.

Holmes et al. (33) suggested that strong and specific stressors to COVID-19 infection could have a profound effect on the mental health of COVID-19 patients. Consistent with mental health experts' primary concern, a considerable number of COVID-19 patients in our study were potentially at a high-risk for mental health problems. Despite the urgent need for mental health research, psychiatric symptoms in COVID-19 patients have not been investigated using validated screening tools (33). Furthermore, most previous studies related to infectious diseases have used retrospective recall methods to assess survivors, however, our data represented the current psychological response to COVID-19. Our findings highlight the need for psychosocial support for the infected patients. Additionally, initial screening for high-risk or vulnerable individuals would be helpful to mitigate long-term adverse consequences. Due to the cross-sectional design and limited information regarding the COVID-19 patients' medical history, our results could not confirm causal-relationship between COVID-19 and mental health problems. However, COVID-19 experiences can constitute a traumatic event beyond a mere stressor. Furthermore, COVID-19 invades the central nervous system, impacting the individual's mental health (34, 35).

There are several limitations to this study. First, as the number of subjects in the dataset is relatively small compared to the total number of confirmed patients in the country, our results may not comprehensively reflect all COVID-19 patients. Additionally, selective biases may have occurred as our sample may have included more individuals who were worried about their mental health problems or those who tend to seek the required help. Additionally, this online assessment collected data only from those who have access to computers or smartphones, therefore, the surveys were responded to by younger participants in the COVID-19 group. Hence, the odds ratio in our analysis cannot be generalized to all the COVID-19 patients. Second, multiple factors could be associated with poorer mental health, however, we only had little information regarding demographic and treatment-related factors. The time interval between the disaster event and mental health assessment, which may affect the severity of mental health symptoms, was not collected in our study. Heterogeneity in time intervals might be greater in the control group than in the COVID-19 group. Additionally, we could not gather sufficient information, such as duration of treatment/quarantine, past psychiatry history, and psychosocial stressors, because our data were collected to provide psychological support to those who needed help, rather than for research purposes. As individuals who experience infectious disease tend to be hesitant to disclose their personal information due to fear of social stigma, minimal demographic and clinical information was collected in the online assessments in this study. Conversely, anonymity would be helpful for screening in communities as this may help confirmed patients respond more honestly.

Despite of these limitations, we carefully emphasize that our findings can provide valuable information regarding the severity of mental health symptoms among COVID-19 patients. This study highlights the need to enhance preparedness regarding mental health support to better manage COVID-19 patients' psychological responses amid the COVID-19 pandemic. Future research should include longitudinal follow-up studies of COVID-19 patients to explore the long-term psychiatric consequences and related risk and protective factors.

## Data Availability Statement

Derived data supporting the findings of this study are available from the corresponding author on request.

## Ethics Statement

The studies involving human participants were reviewed and approved by Institutional Review Board of NCMH. Written informed consent for participation was not required for this study in accordance with the national legislation and the institutional requirements.

## Author Contributions

JL and MS devised the project, the main conceptual ideas, and proof outline. SH, JH, C-HK, and WK contributed to data collection and preparation. SH and JL analyzed data. JL, DL, and MS contributed to the interpretation of the results. JL took the lead in writing the manuscript. All authors provided critical feedback and helped shape the research, analysis, and manuscript.

## Conflict of Interest

The authors declare that the research was conducted in the absence of any commercial or financial relationships that could be construed as a potential conflict of interest.
